# Understanding patient experience of distal tibia or ankle fracture: a qualitative systematic review

**DOI:** 10.1302/2633-1462.43.BJO-2022-0115.R1

**Published:** 2023-03-15

**Authors:** Nathan A. Pearson, Elizabeth Tutton, Stephen E. Gwilym, Alexander Joeris, Richard Grant, David J. Keene, Kirstie L. Haywood

**Affiliations:** 1 Warwick Research in Nursing, Warwick Medical School, University of Warwick, Coventry, UK; 2 Kadoorie, Oxford Trauma and Emergency Care, Nuffield Department of Orthopaedics, Rheumatology and Musculoskeletal Sciences, University of Oxford, Oxford, UK; 3 Major Trauma Centre, Oxford University Hospitals NHS Foundation Trust, Oxford, UK; 4 AO ITC, Clinical Science, AO Foundation, Strettbacherstrausse 6, 8600, Dubendorf, Switzerland; 5 National Institute for Health Research, Applied Research Collaboration, West Midlands; Warwick Medical School, User Teaching and Research Action Partnership; Fragility Fracture Network; Nuffield Department of Orthopaedics, Rheumatology and Musculoskeletal Sciences, University of Oxford, Oxford, UK; 6 Faculty of Health and Life Sciences, University of Exeter, Exeter, UK

**Keywords:** Qualitative, Interviews, Systematic review, Ankle fracture, Patient experience, ankle fractures, distal tibia, clinicians, knees, anxiety, ankle, lower-limb fractures, Strengths, lower limb injury, immobilization

## Abstract

**Aims:**

To systematically review qualitative studies of patients with distal tibia or ankle fracture, and explore their experience of injury and recovery.

**Methods:**

We undertook a systematic review of qualitative studies. Five databases were searched from inception to 1 February 2022. All titles and abstracts were screened, and a subset were independently assessed. Methodological quality was appraised using the Critical Appraisal Skills Programme (CASP) checklist. The GRADE-CERQual checklist was used to assign confidence ratings. Thematic synthesis was used to analyze data with the identification of codes which were drawn together to form subthemes and then themes.

**Results:**

From 2,682 records, 15 studies were reviewed in full and four included in the review. A total of 72 patients were included across the four studies (47 female; mean age 50 years (17 to 80)). Methodological quality was high for all studies, and the GRADE-CERQual checklist provided confidence that the findings were an adequate representation of patient experience of distal tibia or ankle fracture. A central concept of ‘being the same but different’ conveyed the substantial disruption to patients’ self-identity caused by their injury. Patient experience of ‘being the same but different’ was expressed through three interrelated themes, with seven subthemes: i) being proactive where persistence, doing things differently and keeping busy prevailed; ii) living with change including symptoms, and living differently due to challenges at work and leisure; and iii) striving for normality, adapting while lacking in confidence, and feeling fearful and concerned about the future.

**Conclusion:**

Ankle injuries were disruptive, draining, and impacted on patients’ wellbeing. Substantial short- and longer-term challenges were experienced during recovery. Rehabilitation and psychosocial treatment strategies may help to ameliorate these challenges. Patients may benefit from clinicians being cognisant of patient experience when assessing, treating, and discussing expectations and outcomes with patients.

Cite this article: *Bone Jt Open* 2023;4(3):188–197.

## Introduction

This study provides a synthesis of qualitative papers of patient experience of distal tibia or ankle fracture. It aims to provide evidence for practice, direction for future research and to contribute to the development of a core outcome set for ankle fracture.^1^ The addition of patient experience data in the development of a core outcome set helps to ensure consideration of outcomes that are important to patients.^2^

Distal tibia and ankle fractures are a common injury, responsible for up to 14% of hospital fracture admissions in England.^3^ Such fractures are associated with pain, reduced function, and prolonged periods of recovery.^4^ Patient perspectives identify the physical and emotional impact of injury, with some detailing the longer-term consequences.^5-8^ Other qualitative studies of lower-limb fractures identify a loss of self-identity, disempowerment, fragility, and reduced functional ability.^9,10^ Older people note the arduous nature of recovery and high level of support required.^11^ In addition, quantitative studies identify psychosocial factors, depression and anxiety as contributing to poorer outcomes following injury.^12,13^ These studies indicate that recovery from lower limb injury impacts all areas of life.

Synthesizing existing evidence of recovery from distal tibia or ankle fracture will enable a richer, detailed interpretation of specific issues, experience, or circumstance that extends beyond the contributions that a single primary study can provide.^14^ This was identified in osteoporosis where the concept of ‘biographical fracture’, demonstrated substantial changes in self-confidence, self-perception, social living, and levels of anxiety.^15^ The purpose of this systematic review is to appraise qualitative studies of patients with distal tibia or ankle fracture to explore their experience of injury and recovery.

## Methods

This review was registered with PROSPERO (CRD42020182887; protocol included). Review reporting has followed the Enhancing Transparency in Reporting the synthesis of Qualitative research (ENTREQ) statement.^16^

### Patient and public involvement

A group of three Patient and Public involvement (PPI) partners helped to shape the research question and were involved throughout the study. The group met four times with the research group (NAP, ET) to discuss study progress and reviewed the developing themes for resonance with their own experience. One PPI partner (RG) is a co-author on this paper.

### Search strategy

A comprehensive search was conducted to identify all published qualitative studies exploring the lived experiences of people who had sustained a distal tibia and/or ankle fracture. The search strategy combined Medical Subject Headings (MesH) and free text terms (Supplementary search i). The search captured: 1) anatomy-specific (e.g. distal tibia, ankle); 2) fracture-specific; and 3) qualitative terminology (e.g. interview, focus group). Five databases were searched: Medline (OVID), EMBASE (OVID), PsycINFO (OVID), Cumulative Index of Nursing and Allied Health Literature (CINAHL), and Allied and Complimentary Medicine Database (AMED). Searches were conducted from inception until 1 February 2022. Citations of included articles were screened by one reviewer (NAP).

One reviewer (NAP) independently screened all titles and abstracts, and those considered for full text review. A second reviewer (ET) independently doubled-assessed a 10% subset, as suggested in prior systematic reviews.^17^ Any disagreements were resolved through discussion.

### Eligibility criteria

Studies were included if they used qualitative research methods to explore patient experience and were full-text, and English language articles published in peer-reviewed journals. Mixed method studies were included if the qualitative component was separately analyzed and reported. Studies were excluded if they: 1) only reported quantitative methods; 2) did not report qualitative findings separately; and 3) were abstracts, conference proceedings, or editorials.

### Data extraction

Two reviewers (NAP, ET) independently extracted data into a standardized form which included: author and year of publication, study aim(s) and/or research question(s), study design, sample (including size and demographic details), data collection and analysis methods (e.g. thematic analysis), results (including supporting quotes), and study conclusions. A third reviewer (KLH) was available to resolve potential disagreements.

### Quality assessment

Three reviewers (NAP, ET, and KLH) independently evaluated study methodological quality of included studies using the Critical Appraisal Skills Programme (CASP), a ten-item checklist to assess methodological quality of qualitative studies.^18^ Any disagreements were resolved through discussion. One researcher (ET) abstained from assessing one study due to their involvement in the research.

### Data analysis

This qualitative thematic synthesis explored the meanings within the findings and discussion of the included papers.^19,20^ It drew on subtle realism,^21^ where the experience of ankle fracture was considered independent from the researchers but, through interpretation, a new understanding of patient experience of recovery from ankle fracture could be gained. The researchers were reflexive in their approach to interpretation^22^ and discussed their own positionality in relation to their social, cultural and professional context. Throughout analysis discussions with researchers and patient partners explored the similarities and differences across the papers and differing perspectives within the team. The data from the findings and discussion were imported into the software package NVivo 12 (QRS International, USA) to help with data management.

Data were analyzed using a three-step thematic synthesis: 1) line-by-line coding of findings and discussion per included study; 2) development of descriptive themes from the codes produced per study; and 3) generation of analytical themes to draw the emerging descriptive themes together.

One reviewer (NAP) conducted the line-by-line coding. Groups of sentences that conveyed meaning were labelled with a code such as, ‘pain is intrusive in daily life’. Emerging codes were discussed with a second reviewer (ET). Codes with similar meaning were drawn together into descriptive subthemes such as, ‘living with symptoms’. Descriptive subthemes with similar meanings were joined to become analytic themes such as, ‘living with change’. The team worked collaboratively to derive the analytical themes. Rigour drew on trustworthiness,^23^ which involved immersion in the data and clarity in the description of the research process to enable the reader to make judgements about transferability of the findings to other populations.

The Grading of Recommendations Assessment, Development and Evaluation Confidence in Evidence from Reviews of Qualitative research (GRADE-CERQual)^24^ was applied to assess confidence in the ability of the review to provide a reasonable representation of the experience of distal tibia and ankle fracture. Confidence ratings (high, moderate, low, very low) were assigned to: i) methodological limitations,^25^ determined with the CASP checklist;^18^ ii) coherence;^26^ iii) adequacy;^27^ and iv) relevance.^28^ Three reviewers (NAP, ET, KLH) discussed the potential grades to ensure rigour and transparency.

## Results

**Fig. 1:**
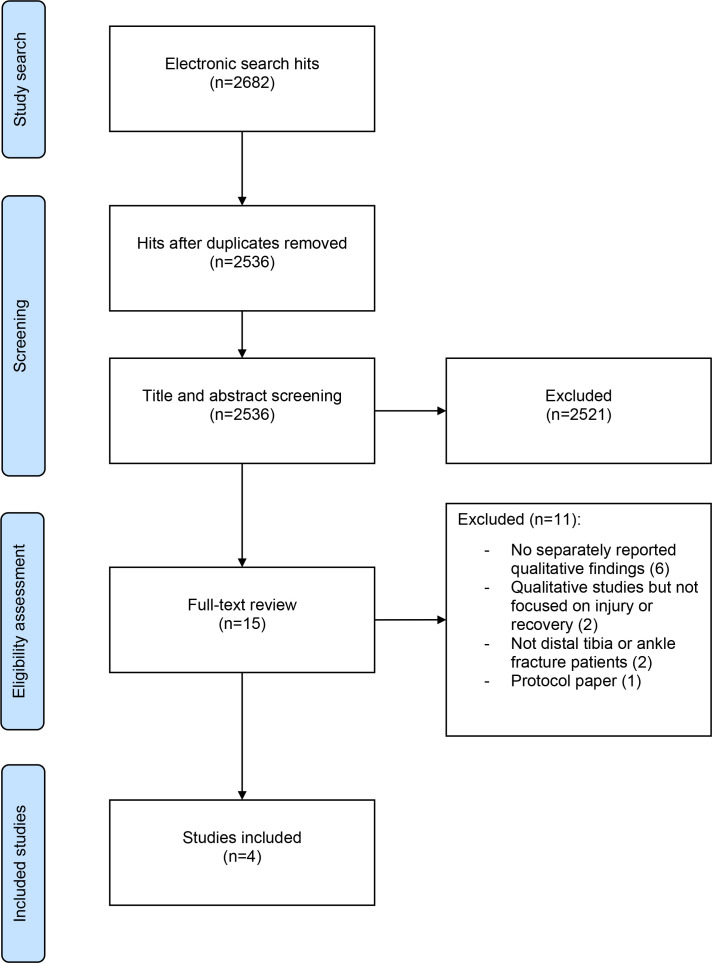
Flowchart of study screening process.

The process of study identification and review are summarized in a Preferred Reporting Items for Systematic review and Meta-Analysis (PRISMA) flow diagram.^29^ From 2,682 records, 15 full text articles were reviewed; four were included in the review (Figure 1).^5-8^ The primary reason for study exclusion was the use of interviews to administer questionnaires (Supplementary table ii).

The four studies included a total of 72 patients (17 to 80 years, mean age of 50 years) following a distal tibia or ankle fracture (47 were females, and 12 were 17 to 30 years, with a mean age of 24 years) (Table I). In addition, six healthcare professionals were included in one study,^5^ but themes were reported separately from the patients, enabling study inclusion. All studies used semi-structured interviews, but differed in their methodological and analytical approaches, adopting phenomenology,^7^ content analysis,^8^ and thematic analysis.^5,6^

**Table I.** Study characteristics.

Author (year)	Country	Fracture type	Sample size, n	Timeframe of interviews	Sex	Mean age, yrs (range)
Jensen (2021)^8^	Denmark	Ankle fracture	14	Within 10 days of injury, and then after 6 weeks	5 M 9 F	45.6 (17 to 72)
Keene (2016)^7^	UK	Closed unstable ankle fracture	36	6 to 10 weeks after treatment	9 M 27 F	67 (60 to 80)
McKeown (2020)^6^	UK	Closed ankle fracture	10	19 to 23 weeks following injury	5 M 5 F	51.6 (21 to 75)
McPhail (2012)^5^	Australia	Distal tibia and/or distal fibula	12 (and 6 healthcare professionals)	6 weeks to over 2 years	6 M 6 F	35.2 (19 to 58)

Study methodological quality was strong (Supplementary table iii). Strengths included clear aims, appropriate methodological approaches, research design and recruitment, data analysis and collection, and clear reporting of findings including their meaning. One study provided partial information on the process of coding and theme derivation.^6^ Two studies provided partial information about the relationship between researcher and participants.^7,8^

### Thematic synthesis

**Fig. 2:**
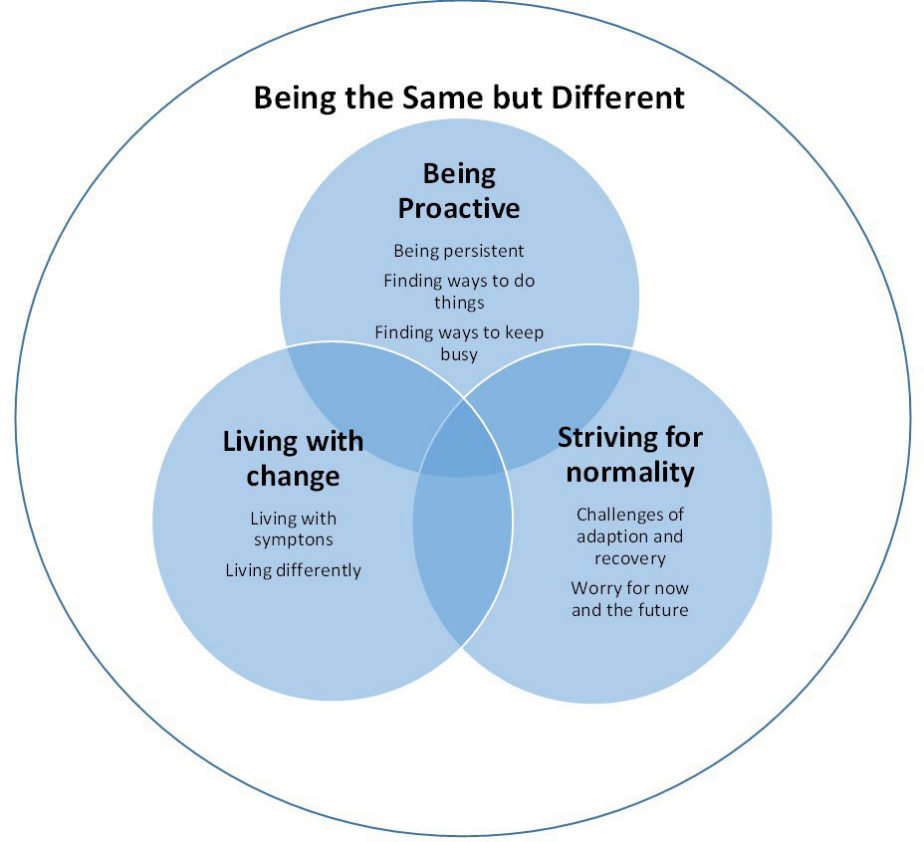
A representation of the analytic themes and descriptive subthemes.

The findings of the review highlight a central concept of ‘being the same but different’, which conveyed the disruption to participants’ sense of self and daily life as they moved forward towards recovery. This was reflected in three analytic themes: 1) being proactive, with subthemes of being persistent, finding ways to do things and finding ways to keep busy; 2) living with change, with subthemes of living with symptoms and living differently; and 3) striving for normality with subthemes of challenges of adaption and recovery, and worry for now and the future. Each of the themes is outlined in Figure 2 and defined in Table II, alongside exemplar supporting evidence and quotes.

**Table II.** Analytical themes and descriptive subthemes of patient experience of distal tibia or ankle fracture.

Analytical theme with definition	Descriptive subtheme with definition	Examples of extracts from the articles and patient quotes with reference to the article (Extracts are from the study findings and discussion;^5-8^ patient quotes, including participant number, are used as supporting evidence)
**Being proactive** The strength of personal attributes and social connections that were essential to sustain recovery	**Being persistent** Keeping going, despite challenges, worries and concerns	Despite needing to rely on others for some things, people also described adapting the way they did things in order to retain their independence as much as possible.^6^ Learning occurred through experience and trial and error, with participants working out what they could and couldn’t manage.^7^ For many, there was a sheer determination to maintain household standards, “It’s a representation of me” (participant 26), even though it was difficult to do and took longer than usual.^7^ At the time of the second interview, this pragmatic attitude had shifted and reality had been different than expected. In particular, patients treated surgically had lost some of their positivity and pragmatism toward future plans.^8^
	**Finding ways to do things** Adapting approaches to activities and tasks to enable them to be completed	“You have to start being imaginative and do things. You just have to learn how am I going to go to the toilet and bathroom. How am I going to wash my hair, how am I going to shower … You felt like you had achieved something if you could do it and you had thought that process through and oh I can do this, I can do that” (participant 22).^7^ When discussing personal care and washing and dressing, many people spoke of finding new routines and adapting to new ways of doing things. Individuals discussed these in relation to their walking aids and weight-bearing status, stating that these factors meant that the process of washing and dressing took much longer.^6^ Many patient participants stated they could still do aspects of their previous activities after sustaining the ankle fracture, but needed to alter these as a result of the fracture. For example, one participant was still able to go swimming, but was not able to use a flipper since the fracture.^5^
	**Finding ways to keep busy** Changing activities to remain active during the non-weight bearing phase	The participants felt that their lives had suddenly lost any sense of freedom and spontaneity. They had to adapt to new activities or bring to the fore existing activities that could be undertaken within a confined environment. Participants who could draw on skills such as sewing, embroidery or computer-based work seemed to fare better in finding suitable occupation. However, planning was required even with these activities if they were to be maintained effectively.^7^
**Living with change** The disruption to daily life as participants learnt to live with symptoms and get on with everyday life	**Living with symptoms** The difficulties of living with the symptoms of an ankle fracture	Individuals spoke of troublesome symptoms around their ankle to varying degrees, including pain, skin changes, wound issues, swelling, reduced movement, and loss of strength and muscle bulk.^6^ “Erm I think the loss of sleep was the worst. Yeah…it wasn’t it wasn’t even so much the pain itself as the fact that I wasn’t sleeping properly and I was tired all the time for a few weeks I think that was the worst” (participant 04).^6^ Struggling to move could be seen as ‘the struggling body’ which left them tired and lacking in energy, and they often felt low and depressed; sunshine, visitors and exercise were good for their spirits.^7^ Impacts in this theme were not limited to the immediate post-fracture period: “it was (many) months before I got the movement back” (participant 10).^5^ Pain influenced patients’ general mobility, yet they were very pragmatic about it at the initial interview, stating that this was expected and acceptable. By the six-week interview, pain was perceived as more intrusive in their everyday lives than initially expected.^8^
	**Living differently** Changed ways of living to accommodate the impact of living with an ankle fracture	The suddenness and unexpected nature of the fracture impacted on participants’ lives in terms of how they felt about themselves as being vulnerable and older people, what they could do, their roles and relationships.^7^ Many people described how others took on the majority of the housework and caring for any dependents.^6^ For those who worked, many people spoke of needing to reduce working hours or be off work due to their injury. Those who were able to work from home discussed doing so throughout the recovery period.^6^ Almost all participants interviewed described a heightened awareness of their ankle, especially when discussing being out in public spaces, walking on uneven surfaces or returning to physical activity.^6^ Prior to the injury, all patients had been active and independent in their everyday lives. They expressed worry and felt uncomfortable being dependent on others, both in their daily living activities in their own homes and also when needing help for shopping or transportation, for instance by car.^8^
**Striving for normality** The difficulties people face when adapting to new ways of living, and their anxiety about whether they will ever really recover	**Challenges of adaption and recovery** The challenges of adapting to having an ankle fracture and the process of recovery	There was a general lack of confidence, fear of falling, or ability to prevent a fall, in relation to moving forward. Moving forward was something participants wanted to do but their bodies were fragile and they were now more watchful and protective, not wishing to cause any further harm.^7^ [discussing using walking aids] “You couldn’t go more than a hundred… two hundred metres without stopping because it just puts so much pressure on your hands” (participant 10).^6^ One individual described an emotional lability during their recovery period, explaining how they would cry a lot more readily than usual throughout this time.^6^ Most participants stated that negative feelings resolved as they were able to return to activities undertaken prior to their ankle fracture.^5^ “The first few weeks I hardly went in the shower because I did not want to sit there and feel helpless” (participant 11).^8^
	**Worry for now and the future** Concerns about living with, and the future implications of having an ankle fracture	“There is worry, yes of course, and all the time you’re slightly worried, is it ever going to be the same again …” (participant 36).^7^ Several people described an anxiety regarding the long-term function of their ankle.^6^ A participant who had experienced a difficult recovery after fracturing his ankle 18 months earlier stated he tended to “feel quite depressed and down a lot, and dwell on what happened and keep replaying things in (his) mind” (participant 10).^5^ Some participants reported ongoing unresolved anxiety or depression months after plaster removal.^5^ Several patients also expressed that they were more worried than they had initially expected. Again, worries related to how the injury would influence their working conditions or their ability to return to work were especially predominant.^8^

## Theme 1

Being proactive conveyed the strength of personal attributes and social connections that were essential to sustain recovery. It consisted of three subthemes which reflect: i) being persistent; ii) finding ways to do things; and iii) finding ways to keep busy.

### i. Being persistent

Being persistent reflected the mind-set of participants who were determined to return to and continue living their lives, despite the new challenges they faced. They were determined to regain independence and learnt through experience the extent of their own capability. Feelings of vulnerability were mitigated through keeping positive, getting back to work, and engaging with their friends.

“…for mental reasons it’s good to get back to normal errm that was quite important for me to feel as though I was able to take charge of my own life again. Erm yeah rather than relying on other people” (Participant 9)*.*^6^

### ii. Finding ways to do things

Normal roles and activities were disrupted. This subtheme captured the creative and practical steps participants took to facilitate engagement in activities when non-weightbearing. These included rethinking how to wash and dress, role reversal with a partner, and modifying activities to reduce the ‘demand’ on the ankle. This process was frustrating, but also satisfying as they could maintain some normality in their everyday life.

“Erm yes I discovered that if I got on my knees…hands and knees (pause) I could actually…not hoover…but I could get a stick brush and sweep the carpet. Erm I did that for quite a while. And then you had to make sure that you were near something so that I could get back up again. I also did gardening on my hands and knees because it was…to see it just going…it was heartbreaking. And I thought ‘right if I could get out there I’m sure if I’d got something to kneel on I can actually do that’ and I did” (Participant 7).^6^

### iii. Finding ways to keep busy

There was a loss of purposeful occupation, freedom and spontaneity. Mental and practical adjustments were taken to overcome their reduced leisure or work activities, such as childcare, cycling, and woodwork. New less physically demanding activities, such as sewing and computer-based activities, were undertaken. Social connections were key to supporting them through these adjustments.

“So you just put yourself in a completely different zone, I can’t describe it, it’s not like, I don’t mean like meditating or something but your life becomes very small and within that you try to control it as much as possible I think, in terms of your comfort, your entertainment, you know all that stuff … It is only now that I think that yes that is what I was doing, I was in a different layer of existence – a parallel universe almost. I had switched off from everything normal and if I did do anything normal, it was a bonus, like wheelchair gardening” (Participant 12).^7^

## Theme 2

Living with change conveyed the disruption to daily life as participants learnt to live with symptoms and get on with everyday life. This theme consists of two subthemes which capture: i) living with the symptoms; and ii) living differently.

### i. Living with symptoms

The consequences of treatment were difficult and challenging for participants. Physically, participants reported difficulty with needing to ‘hop’ to get around. Pain created problems when trying to get to or stay asleep affecting their mood and performance. Some participants identified low mood or feeling depressed. Symptoms identified by participants, such as reduced muscle strength, range of motion, swelling, pain, altered sensations, and discomfort, could affect the pace and distance the participant could walk.

“… but the pain has actually been enormous, and it has worried me a bit, and I actually started to convince myself that it is broken higher up on the leg because it is up there the pain sits … it is actually not down where it is broken [in the ankle]” (Participant 3).^8^

### ii. Living differently

All aspects of life were affected. In addition to the early struggles when less mobile, limitations when working could affect income. Sedentary roles were more likely to be manageable and often light or modified duties were sought. Many felt they could no longer socialize as normal, and some felt they were a burden on family and friends.

“Yes, it’s changed his life as well as mine. And as for one’s sex life? What’s that! I think he’s almost been afraid to touch me as though I have become fragile because some bones have broken. I think I’m not going to break you know, it’s just my ankle everything is absolutely fine but he’s obviously worried, he’s concerned he’s lacking in confidence about it but who knows how long it will take to repair before it all starts to feel normal again” (Participant 7).^7^

## Theme 3

Striving for normality conveyed the challenge participants have when adapting to new ways of living, and their anxiety about whether they will ever really recover. Striving for normality is identified through two subthemes: i) challenges of adaption and recovery; and ii) worry for now and the future.

### i. Challenges of adaption and recovery

Movement was difficult with walking aides considered challenging to use and causing pain in hands and shoulders. Navigating stairs was not easy and some found it safer to ascend sitting down. Participants felt vulnerable, had reduced energy and could feel tired. They also lacked confidence, slowed down and exercised greater care and caution. Recovery required navigation and adaptions enabled participants to live the fullest life possible.

“It was more effort to do anything, I felt so tired all the time” (Participant 8).^5^

“I must say, I have really felt handicapped in ways that DO matter” (Participant 12).^8^

### ii. Worry for now and the future

Participants felt anxious about having an ankle fracture, the long-term implications and when undertaking activities of daily living or leisure. There was some indication that older people were concerned about falling while younger people were anxious about sporting and leisure activities. Regaining independence through being able to walk and drive were imperative. Acceptance was one way for people to contain their emotions and keep going but concerns about returning to ‘normal’ remained.

“…and that is so important to me to be able to get back to that…to be fit you know so I’m quite weight conscious. I’m very conscious of the fact that I don’t want to get fat sitting around and not doing anything” (Participant 5).^6^

### Assessing confidence in review findings (GRADE-CERQual assessment)

The review provided moderate confidence in its ability to reflect patients’ experience of distal tibia or ankle fracture due to the low number of studies. (See Table III; Supplementary table iv). However, confidence was evidenced by high methodological quality, providing rich and detailed data, which demonstrated similarity of patient experience across the studies. A category from a single study, ‘finding ways to keep busy’ was rated as ‘low confidence’ (Table III). This finding contributed to understanding how patients kept occupied when non-weightbearing following injury, while they are limited in their usual pursuits.

**Table III.** Summary of qualitative findings with GRADE-CERQual assessments of confidence (also see Supplementary table iv).

Summary of review finding	References for studies contributing to the finding	Assessment of confidence
**Theme 1: Being proactive**		
Being persistent: Keeping going, despite challenges, worries, or concerns.	^6-8^	Moderate confidence
Finding ways to do things: Adapting approaches to activities and tasks to enable them to be completed.	^5-7^	Moderate confidence
Finding ways to keep busy: Changing activities to remain active during the non-weight bearing phase.	^7^	Low confidence
**Theme 2: Living with change**		
Living with symptoms: The difficulties of living with the symptoms of an ankle fracture.	^5-8^	High confidence
Living differently: Changed ways of living to accommodate the impact of living with an ankle fracture.	^5-8^	High confidence
**Theme 3: Striving for normality**		
Challenges of adaption and recovery: The challenges of adapting to having an ankle fracture and the process of recovery.	^5-7^	High confidence
Worry for now and the future: Concerns about living with, and the future implications of having an ankle fracture.	^5-8^	High confidence

## Discussion

This qualitative evidence synthesis of patient experience of fractures of the distal tibia and ankle identified an overarching theme of ‘being the same but different’. This was conveyed through three themes: 1) being proactive, which embraced the strength of personal attributes and social connections essential to sustain recovery; 2) living with change, which captured the disruption to daily life, how people coped with symptoms and got on with life; and 3) striving for normality, which reflected the challenges of adaption, anxiety, and concerns about recovery. The substantial physical, emotional, and social impact of ankle fracture, which can extend over the longer-term, is evident. Implications for practice include evaluation of early weightbearing, assessing the impact of rehabilitation, and consideration of psychological and social support to sustain mental wellbeing.

Early recovery while non-weightbearing was a challenging time. Home and work environments were not conducive to living with a walking disability and dependency on others disrupted established roles and relationships. Creative ways were found to manage daily life, but many activities were put on hold as in other injuries of the lower limb.^9-11,30^ The requirement for non-weightbearing for patients who have surgery for ankle fracture is being challenged.^31^ If supportive evidence is found, this may ease the burden of disability in early recovery for this group of patients.

Living with change identified the struggle to manage a changed body, including the experience of symptoms, such as pain, stiffness, and swelling. Managing pain can be difficult after injury and health professionals can underestimate pain.^32^ Pain with neuropathic characteristics can also be present but not necessarily treated.^33^ Loss of muscle strength and flexibility was also a concern, and an ongoing study is exploring the impact of progressive exercise versus advice after ankle fracture treated with at least four weeks of immobilization.^34^

Striving for normality was hard work, participants had mixed success with mobility aides and lacked confidence walking. Approaching activity with a sense of caution, fear of falling and causing further injury was similar to older people with hip fracture.^30^ Challenges of lack of mobility, getting back to working and social participation, and weight gain due to reduced mobility could influence mood. This is also evident in other lower limb fractures.^9,10^ Ongoing anxiety and low mood due to reduced confidence and the uncertainty of recovery, may reduce resilience to sustain rehabilitation over time. Strategies to address this may help reduce the impact on longer term frailty in older people, or the return to sports in younger adults.

### Strengths and weaknesses

The strength of the review is that it included patients with a wide range of ages. However, only 12 participants were younger (aged 17 to 30 years). Recovery up to two-years post-injury, and both distal tibia^5^ and ankle fracture,^5-8^ were included. There were, however, a limited number of patients with a distal tibia injury and the studies were all from healthcare in high income countries. Further qualitative research in this patient group and in low-income settings may increase understanding of the challenges of recovery.

In conclusion, this systematic review highlights the lack of research in patient experience of recovery from distal tibia and ankle fracture, particularly from distal tibia and in younger adults. Despite this, the review highlights similarities across studies exploring the impact of fractures affecting the lower limb. Further research is ongoing that will explore early weightbearing and rehabilitation, but strategies for social and psychological support may also be needed. Further research is needed to identify strategies for self-management and to identify those at risk of poor recovery to prioritize allocation of resources. Surgeons and multidisciplinary teams may benefit from being aware of the longer-term uncertainty of full recovery and its impact on patients’ lives.

**Take home message**

- This systematic review highlighted a paucity of qualitative research focused on patient experience of recovery from distal tibia or ankle fracture.

- Studies included adult patients aged 17 to 80 years; however, there were very few patients aged 16 to 30 years included in the studies, despite the fact this age group is often affected.

- Patient experience of distal tibia or ankle fracture highlights their challenges and the longer-term nature of recovery for this group.

## References

[1] Pearson NA Tutton E Joeris A et al. Co-producing a multi-stakeholder Core Outcome Set for distal Tibia and Ankle fractures (COSTA): a study protocol Trials 2021 22 1 443 34247628 10.1186/s13063-021-05415-1 PMC8273034

[2] Haywood KL Pearson N Morrison LJ Castrén M Lilja G Perkins GD Assessing health-related quality of life (HRQoL) in survivors of out-of-hospital cardiac arrest: A systematic review of patient-reported outcome measures Resuscitation 2018 123 22 37 29191703 10.1016/j.resuscitation.2017.11.065

[3] Jennison T Brinsden M Fracture admission trends in England over a ten-year period Ann R Coll Surg Engl 2019 101 3 208 214 30698459 10.1308/rcsann.2019.0002 PMC6400910

[4] Ribeiro de Ávila V Bento T Gomes W Leitão J Fortuna de Sousa N Functional outcomes and quality of life after ankle fracture surgically treated: a systematic review J Sport Rehabil 2018 27 3 274 283 28338395 10.1123/jsr.2016-0199

[5] McPhail SM Dunstan J Canning J Haines TP Life impact of ankle fractures: qualitative analysis of patient and clinician experiences BMC Musculoskelet Disord 2012 13 1 224 23171034 10.1186/1471-2474-13-224 PMC3517753

[6] McKeown R Kearney RS Liew ZH Ellard DR Patient experiences of an ankle fracture and the most important factors in their recovery: a qualitative interview study BMJ Open 2020 10 2 e033539 10.1136/bmjopen-2019-033539 PMC7044932 32024789

[7] Keene DJ Mistry D Nam J et al. The Ankle Injury Management (AIM) trial: a pragmatic, multicentre, equivalence randomised controlled trial and economic evaluation comparing close contact casting with open surgical reduction and internal fixation in the treatment of unstable ankle fractures in patients aged over 60 years Health Technol Assess 2016 20 75 1 158 10.3310/hta20750 PMC5075748 27735787

[8] Jensen CM Serritslev R Abrahamsen C Patients perspective on treatment and early rehabilitation after an ankle fracture: A longitudinal qualitative study Int J Orthop Trauma Nurs 2022 46 100916 34802956 10.1016/j.ijotn.2021.100916

[9] Trickett RW Mudge E Price P Pallister I A qualitative approach to recovery after open tibial fracture: the road to a novel, patient-derived recovery scale Injury 2012 43 7 1071 1078 22356720 10.1016/j.injury.2012.01.027

[10] Rees S Tutton E Achten J Bruce J Costa ML Patient experience of long-term recovery after open fracture of the lower limb: a qualitative study using interviews in a community setting BMJ Open 2019 9 10 e031261 10.1136/bmjopen-2019-031261 PMC6797425 31601595

[11] Phelps EE Tutton E Griffin X Baird J Arc T A qualitative study of patients’ experience of recovery after a distal femoral fracture Injury 2019 50 10 1750 1755 31371167 10.1016/j.injury.2019.07.021

[12] Archer KR Abraham CM Obremskey WT Psychosocial factors predict pain and physical health after lower extremity trauma Clin Orthop Relat Res 2015 473 11 3519 3526 26282387 10.1007/s11999-015-4504-6 PMC4586200

[13] Vincent HK Hagen JE Zdziarski-Horodyski LA et al. Patient-reported outcomes measurement information system outcome measures and mental health in orthopaedic trauma patients during early recovery J Orthop Trauma 2018 32 9 467 473 30130305 10.1097/BOT.0000000000001245

[14] Flemming K Noyes J Qualitative evidence synthesis: where are we at? Int J Qual Methods 2021 20 160940692199327

[15] Barker KL Toye F Lowe CJM A qualitative systematic review of patients’ experience of osteoporosis using meta-ethnography Arch Osteoporos 2016 11 1 33 27739032 10.1007/s11657-016-0286-z PMC5063904

[16] Tong A Flemming K McInnes E Oliver S Craig J Enhancing transparency in reporting the synthesis of qualitative research: ENTREQ BMC Med Res Methodol 2012 12 181 23185978 10.1186/1471-2288-12-181 PMC3552766

[17] Pearson NA Packham JC Tutton E Parsons H Haywood KL Assessing fatigue in adults with axial spondyloarthritis: a systematic review of the quality and acceptability of patient-reported outcome measures Rheumatol Adv Pract 2018 2 2 rky017 31431965 10.1093/rap/rky017 PMC6649921

[18] No authors listed Critical Appraisal Skills Programme UK checklists 2020 https://casp-uk.net/casp-tools-checklists/ date last accessed 31 January 2023

[19] Thomas J Harden A Methods for the thematic synthesis of qualitative research in systematic reviews BMC Med Res Methodol 2008 8 45 18616818 10.1186/1471-2288-8-45 PMC2478656

[20] Braun V Clarke V Thematic Analysis: A Practical Guide UK Sage Publications 2021

[21] Hammersley M What’s wrong with ethnography New York Routledge 1992

[22] Rowe WE Positionality In Coghlan D Brydon-Miller M The Sage Encyclopedia of Action Research 2014

[23] Lincoln YS Guba EG Pilotta JJ Naturalistic inquiry International Journal of Intercultural Relations 1985 9 4 438 439

[24] Lewin S Bohren M Rashidian A et al. Applying GRADE-CERQual to qualitative evidence synthesis findings-paper 2: how to make an overall CERQual assessment of confidence and create a Summary of Qualitative Findings table Implement Sci 2018 13 Suppl 1 10 29384082 10.1186/s13012-017-0689-2 PMC5791047

[25] Munthe-Kaas H Bohren MA Glenton C et al. Applying GRADE-CERQual to qualitative evidence synthesis findings-paper 3: how to assess methodological limitations Implement Sci 2018 13 Suppl 1 9 29384078 10.1186/s13012-017-0690-9 PMC5791044

[26] Colvin CJ Garside R Wainwright M et al. Applying GRADE-CERQual to qualitative evidence synthesis findings-paper 4: how to assess coherence Implement Sci 2018 13 Suppl 1 13 29384081 10.1186/s13012-017-0691-8 PMC5791039

[27] Glenton C Carlsen B Lewin S et al. Applying GRADE-CERQual to qualitative evidence synthesis findings-paper 5: how to assess adequacy of data Implement Sci 2018 13 Suppl 1 14 29384077 10.1186/s13012-017-0692-7 PMC5791045

[28] Noyes J Booth A Lewin S et al. Applying GRADE-CERQual to qualitative evidence synthesis findings-paper 6: how to assess relevance of the data Implement Sci 2018 13 Suppl 1 4 29384080 10.1186/s13012-017-0693-6 PMC5791042

[29] Moher D Liberati A Tetzlaff J Altman DG Group P Preferred reporting items for systematic reviews and meta-analyses: the PRISMA statement BMJ 2009 339 b2535 19622551 10.1136/bmj.b2535 PMC2714657

[30] Griffiths F Mason V Boardman F et al. Evaluating recovery following hip fracture: a qualitative interview study of what is important to patients BMJ Open 2015 5 1 e005406 10.1136/bmjopen-2014-005406 PMC4289715 25564138

[31] Bretherton CP Claireaux HA Achten J et al. Protocol for the Weight-bearing in Ankle Fractures (WAX) trial: a multicentre prospective non-inferiority trial of early versus delayed weight-bearing after operatively managed ankle fracture BMC Musculoskelet Disord 2021 22 1 672 34372803 10.1186/s12891-021-04560-7 PMC8353856

[32] Seers T Derry S Seers K Moore RA Professionals underestimate patients’ pain: a comprehensive review Pain 2018 159 5 811 818 29351169 10.1097/j.pain.0000000000001165

[33] Keene DJ Knight R Bruce J et al. Chronic pain with neuropathic characteristics after surgery for major trauma to the lower limb: prevalence, predictors, and association with pain severity, disability, and quality of life in the UK WHiST trial Bone Joint J 2021 103-B 6 1047 1054 33902306 10.1302/0301-620X.103B.BJJ-2020-2204.R1

[34] Keene DJ Srikesavan C Achten J et al. Flexibility and resistance exercises versus usual care for improving pain and function after distal radius fracture in adults aged 50 years or over: protocol for the WISE randomised multicentre feasibility trial Pilot Feasibility Stud 2022 8 1 55 35256000 10.1186/s40814-022-01011-5 PMC8898994

